# *Clostridioides difficile* infection study models and prospectives for probing the microbe-host interface

**DOI:** 10.1128/jb.00407-24

**Published:** 2025-02-06

**Authors:** Tatiana Zvonareva, David S. Courson, Erin B. Purcell

**Affiliations:** 1 Department of Chemistry & Biochemistry, Old Dominion University 6042 , Norfolk, Virginia, USA; University of Illinois Chicago, Chicago, Illinois, USA

**Keywords:** *Clostridioides difficile*, CDI, host-pathogen interface, rose chamber

## Abstract

*Clostridioides difficile* infection (CDI) is an urgent public health threat with a high rate of recurrence and limited treatment options. *In vivo* models have been indispensable in understanding CDI pathophysiology and establishing treatment protocols and continue to be essential in pre-clinal testing. More importantly, *in vivo* models offer the opportunity to probe the complex systemic host response to the microbe, which is impossible to recapitulate *in vitro*. Nonetheless, constraints related to the availability of animal models, cost, ethical considerations, and regulatory control limit their accessibility for basic research. Furthermore, physiological and habitual divergences between animal models and humans often result in poor translatability to human patients. In addition to being more accessible, *in vitro* CDI models offer more control over experimental parameters and allow dynamic analysis of early infection. *In vitro* fermentation offers models for probing microbe-microbe and microbe-microbiome interactions, while continuous multi-stage platforms allow opportunities to study *C. difficile* pathophysiology and treatment in context with human-derived microbiota. However, these platforms are not suitable for probing the host-pathogen interface, leaving the challenge of modeling early CDI unanswered. As a result, alternative *in vitro* co-culture platforms are being developed. This review evaluates the strengths and weaknesses of each approach, as well as future directions for *C. difficile* research.

## INTRODUCTION

*Clostridioides difficile* (*C. difficile*) is an opportunistic, spore-producing, Gram-positive anaerobe inhabiting the lower gastrointestinal (GI) tract of humans and animals (1–3). This bacterium is the leading cause of infective, healthcare-associated diarrhea in the US, and is also linked to community-associated colitis (4, 5). The clinical manifestations of *C. difficile* infection (CDI) range from a asymptomatic intestinal colonization or mild, self-mitigating GI disturbance to abdominal pain and fever, accompanied by severe, persistent diarrhea (6). Patients with advanced CDI may develop pseudomembranous colitis (PMC), paralytic ileus, toxic megacolon, perforation of the colon, and even sepsis (1–3). CDI affects up to half a million Americans every year, with elderly, female, and immunocompromised patients being particularly susceptible to this infection (5, 7). The annual CDI-related death toll in the US reaches up to 30,000 lives with an estimated inpatient healthcare cost of $5B (7). Since 2013, the United States Centers for Disease Control and Prevention (CDC) has classified CDI as an “urgent public health care threat,” emphasizing the need for the immediate development of treatment and prevention strategies necessary to contain this healthcare crisis (3).

CDI recurrence poses the main challenge in the treatment of the disease and results predominantly from the bacterium’s ability to sporulate and form biofilms (8–11). Transformation into a metabolically dormant spore is an essential survival mechanism utilized by *C. difficile* to overcome environmental stress, including exposure to oxygen (12, 13). The complex peptidoglycan composition of the spore coat is impermeable to most hospital-grade disinfectants and low-pH environments (14–16). Ingested spores can remain within hosts as reservoirs for opportunistic re-colonization followed by antibiotic-mediated disruption of the intestinal microbiome (17) or spread to new hosts by settling on hospital surfaces (18), water bodies, food, and in soil (19). Spores remain dormant within the GI tract unless activated by the primary bile acid cholate and its conjugates, glycocholate, and taurocholate (20, 21). Metabolism by commensal bacteria in the ileum converts these pro-germination bile acids into the secondary bile acids deoxycholate and lithocholate, which inhibit spore germination and *C. difficile* outgrowth (22). Commensal bacteria in the host microbiota thus modulate the balance of primary and secondary bile acids, contributing to *C. difficile* colonization resistance (23). Exposure to broad-spectrum antibiotics disrupts the innate microbiota, reducing the rate of cholate turnover and increasing the ratio of primary to secondary bile acids, which facilitates spore germination into metabolically active, toxin-producing *C. difficile* cells (17).

*C. difficile* toxins target host epithelial cells, causing irreversible changes in the cytoskeleton that ultimately lead to cell death, inflammation, and diarrhea (24). As colonization progresses, *C. difficile* continues to ensure its survival by adhering to the intestinal mucosa as a biofilm. *C. difficile* biofilm contains an extracellular matrix composed of polysaccharides, proteins, and extracellular DNA (9). Biofilms are believed to enhance *C. difficile* oxygen tolerance and protect the vegetative cells from antibiotics, attacks by the host immune system, and inhibitory metabolites produced by competing bacteria (25–27). Biofilm-mediated antibiotic protection may contribute to increasing the virulence of the epidemic strains, although this area requires further research (28).

Since the 1970s, antibiotic therapy has been the primary weapon of choice against this pathogen (3). For over three decades, vancomycin and metronidazole were the front-line antibiotic therapies used in the treatment of CDI, but their effectiveness has declined over time (29–31). In 2011, fidaxomicin became the first FDA-approved *C. difficile*-specific antimicrobial agent for the treatment of recurrent CDI (32). Despite its success in reducing the rate of relapse in patients, widespread administration of this drug is limited due to cost and/or lack of availability in certain regions of the world (33, 34). Furthermore, the development of resistance is a perpetual issue. Although not yet numerous, reports of fidaxomicin-resistant strains have begun to emerge, both *in vitro* and *in vivo* (35, 36).

Given the limitations of conventional antibiotic therapy against CDI, other treatment strategies are being explored. Microbiota restoration methods are gaining attention, including fecal microbiota transplantation (FMT) (37, 38). FMT is the first microbiota-based oral agent FDA-approved for use in patients with recurrent CDI (39). However, the exact therapeutic mechanism of the approach remains to be elucidated as it is unclear whether FMT directly targets *C. difficile in vivo* or modulates its growth by replenishing the population of competing bacteria from the donor microbiome (40). In addition, the long-term effects of microbiota transplant on human health are poorly understood (41), including the risk of infecting patients with other deadly microbes (42). The widespread use of FMT therapy is also limited by resistance from patients reluctant to consume medication derived from another person’s stool (43).

Despite the substantial progress made in our understanding of CDI pathophysiology and treatment, more work is needed for the advancement of conventional antibiotic therapy, and design of novel treatments. The main limitation in the field is the availability of economically and logistically accessible research models compatible with obligate anaerobes. Anaerobic bacterial culture requires specialized equipment, offers no opportunity for co-culture with aerobic host cells, and limits the types of experiments that can be performed as many signal detection methods require molecular oxygen. As a result, a significant amount of *C. difficile* research has been done in animals, which eliminates the need for designing a functional anaerobic *in vitro* platform but is largely inaccessible for many research teams. Both *in vivo* and *in vitro C. difficile* research suffer from a lack of real-time probing of the host-pathogen interface during the early stages of infection, limiting our understanding of *C. difficile* survival strategies and discovery of novel drug targets. Therefore, the future of CDI research depends on achieving a balance between the physiological relevance of the *in vivo* models and the accessibility of the *in vitro* study design in modeling the host-pathogen interactions. This review will consider current progress in the development of research models, address the challenges associated with probing CDI both *in vivo* and *in vitro,* as well as propose future directions for optimizing the effectiveness-to-accessibility balance for probing the host-pathogen interface *in vitro*.

## ANIMAL MODELS

**Fig 1:**
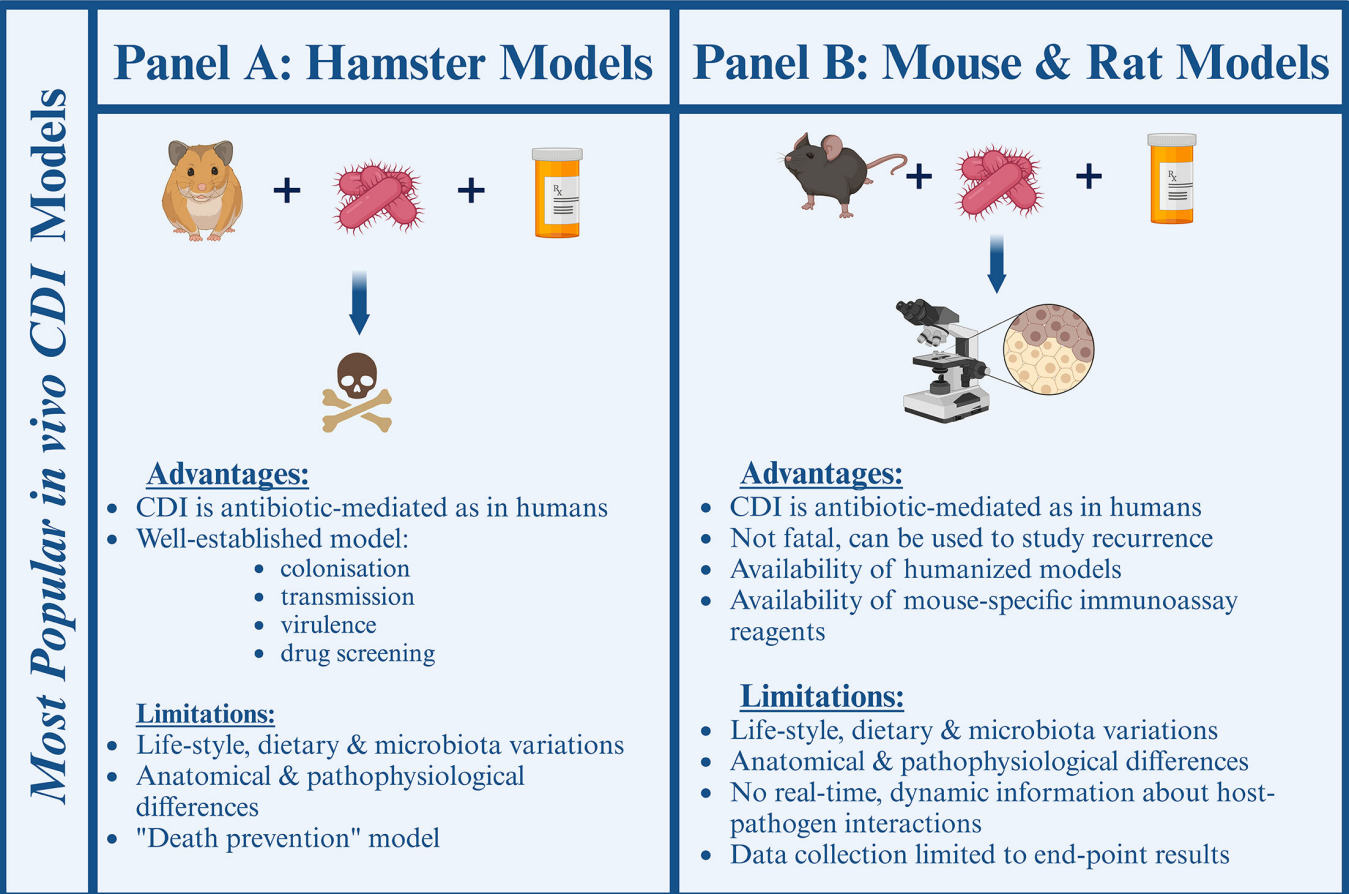
Summary of the most utilized rodent *in vivo* models in CDI research. (**A**) Hamsters are a death-prevention model widely used for drug screening, modeling acute *C. difficile* infection, transmission, population dynamics, and virulence. (**B**) Mice model recurrent infection and host immune response in CDI. Created with BioRender.com

Our current understanding of CDI pathophysiology and development of early treatment protocols would not have been possible without first *in vivo* rodent models (Fig. 1) (44–59). Experiments involving Syrian hamsters linked *C. difficile* to antibiotic-induced enterocolitis and validated hamsters as an early *in vivo* CDI model (44, 45, 48). Hamsters were utilized for studying *C. difficile* transmission (48, 60), colonization (49, 61), virulence (47, 51, 62–64), and population dynamics (49, 65, 66). Similarly to humans, CDI in hamsters is caused by antibiotic-mediated disruption of the intestinal microbiota, followed by colonization of the gut epithelium (48). The pathophysiology of CDI in hamsters involves inflammation and enlargement of the colon and cecum, resulting in reduced intestinal motility and fulminant failure. CDI in hamsters is lethal to animals unless treated with antibiotics capable of removing the infection, effectively making hamsters an *in vivo* death prevention model. This makes hamsters an excellent model for probing novel antimicrobial agents and studying toxin-mediated diseases, but presents challenges when modeling colonization and recurrence due to the animals’ acute sensitivity to *C. difficile* toxins (67–69). In addition, studies of the whole-organism immune response to infection are limited by a general lack of genetically modified animal lines and hamster-specific immunogenic reagents such as species-specific antibodies and cytokine assays. Although hamsters are still an active part of CDI research, the frequency of reports utilizing hamsters has decreased over the years as the scientific community has moved toward discovering more suitable *in vivo* models.

Similarly to hamsters and humans, CDI in mice and rats is allowed by antibiotic-mediated disruption of the host microbiome (70, 71). In contrast to hamsters, mice and rats can recover from the effects of the infection, which allows modeling of infection recurrence. Human-associated mouse models have been utilized in characterizing *C. difficile* pathophysiology, colonization and recurrence (49, 72), virulence (73, 74), bile salt metabolism (17, 75, 76), the role of environmental conditions and population dynamics disease progression (17, 73, 77). Furthermore, mice offer a greater availability of validated, genetically modified animals, including the immunodeficient and immuno-humanized models, making them invaluable for understanding CDI in the context of human microbiome and immunity (78–80). Immuno-humanized mice have been used to evaluate the role of neutrophil recruitment in disease progression and the development of an immunogenic treatment strategy (81–85). Rodent models were used in probing the inflammatory response to *C. difficile* virulence factors through toxin expression (53, 54, 86, 87), spores (12, 20, 53, 87), and the presence of surface proteins (79, 88). Human intestinal xenografts have been successfully transplanted to immunodeficient mice to demonstrate the enterotoxin nature of *C. difficile* toxins A and B on human epithelia (86).

Other mammalian models have been used to contribute to *C. difficile* toxin characterization, including monkeys (89), rats (54), rabbits (56, 90), piglets (91), Guinea pigs (87), and foals (92). Animal models were essential in developing CDI-specific antibiotic treatment protocols. Moreover, the clinical impact of new drug candidates must be tested *in vivo* before being approved for use in humans (93). In addition, animal infections were necessary to assess CDI pathophysiology and treatment in the context of host innate and adaptive immune systems. Unfortunately, the physiological relevance associated with animal research is offset by limited accessibility. Application of most animal models requires the approval of ethical protocols, availability of animal husbandry resources, and extensive funding. As a result, a limited number of researchers are able to utilize these models.

Non-mammalian animal models exhibiting genetic, structural, and functional similarities with human systems provide accessible alternatives to *in vivo* applications. For example, great wax moth larvae (*Galleria mellonella*) are a resource-effective invertebrate model that is gaining popularity (94). *G. mellonella* is not subject to ethical regulation and requires no specialized husbandry equipment or highly trained personnel. Wax moths are commercially available, tolerate a wide range of temperatures including 37°C, and have a fully sequenced genome (95). *G. mellonella* is an established invertebrate bacterial infections model (96–99) that has been used to compare the pathogenicity of clinical *C. difficile* isolates (100, 101), elucidate the role of *C. difficile* anti-sigma factor RsbW in establishing colonization and infection (102), and probe the effectiveness of a phage cocktail in anti-CDI immune therapy (103). However, the research application of these models is limited due to the lack of certified distribution centers operating on standard handling and breeding practices. As a result, *G. mellonella* research outcomes are affected by discrepancies in genetic background and the overall health of commercially purchased organisms. In addition, the limited availability of microarrays and RNA interference libraries significantly hinders the interpretation of *G. mellonella* genetic experiments, and a lack of established fluorescent reporter lines makes real-time detection, including live microscopy, unavailable for this model at this time.

Zebrafish (*Danio rerio*) are another accessible animal model, that, unlike *G. mellonella*, can integrate the complexity of a vertebrate organism with advanced detection methods, including real-time fluorescent microscopy (104–106). These freshwater fish utilize *ex vivo* fertilization of numerous eggs that undergo well-characterized external embryogenesis compatible with light microscopy (107). This allows for high-throughput sibling comparison experiments, valuable for drug screening and toxin screening (108, 109). Conveniently, a substantial number of established and well-characterized cell- and tissue-specific fluorescent reporter zebrafish lines are available for research use from specialized stock centers (110–112). This animal model is adaptable for gene editing, optogenetics, and transcriptomics (111, 113, 114). Zebrafish exhibit notable similarities with mammalian organ composition, regulation, and function, including the GI tract (115, 116). Although the zebrafish intestinal epithelium is less complex in architecture than that of mammals, gene transcription, regulation, and biochemical functionality of GI epithelial cells are conserved (116–118). In addition, innate immunity is highly homologous between humans and zebrafish, including pathogen recognition function and autophagy, and can be modeled in isolation in larvae (119–122). Therefore, zebrafish can be utilized as a model for GI research, and host-pathogen findings produced as a result have a high translatability potential to humans (117).

Zebrafish embryos have been used to demonstrate the accumulation of purified clostridial toxins TcdB (123) and TcdA (124) in organs. A transgenic zebrafish line was also utilized for dynamic analysis of the ventricular response to TcdA (125) and systemic effects of TcdB obtained from various *C. difficile* strains (124). Multiple *C. difficile* infection methods have been utilized for zebrafish, including immersion into bacterial inoculum (118) and microgavage delivery of bacterial load into the GI tract of agar-immobilized larvae (126). Inoculation with microgavage was utilized for probing neutrophil and macrophage recruitment to the infection site using corresponding zebrafish reporter lines. This demonstrated longer persistence of the infection in gnobiotic fish when compared to the wild type (120 hours post-fertilization [hpf] vs 24 hpf), indicating the affinity of the innate zebrafish microbiome to resist *C. difficile* colonization (126). Of note, to our knowledge, there are no reports attempting antibiotic-mediated susceptibility to CDI as is typically done with rodents.

There are, however, drawbacks associated with this *in vivo* model, including the lower body temperature and increased intestinal lumen oxygenation. Nonetheless, this animal model has an immense potential for modeling various aspects of host-pathogen interface in live fish on a whole-organism level. This can be achieved with optically transparent mutant lines (105, 110), crossed with cell- and/or tissue-specific fluorescent reporter lines that have already been established. This type of *in vivo* model design is not only impossible to achieve with rodent models but also involves a fraction of the cost and resources associated with animal husbandry of mice or rats. Considering the limited number of studies attempting to induce CDI in this organism, zebrafish is likely an underutilized CDI model (117).

In addition to cost and logistical challenges, all animal models of human disease have physiological limitations due to interspecies differences. Lifestyle and dietary divergences contribute to variations in commensal microbiota composition, which can affect immune response, host-pathogen interaction, disease progression, as well as drug delivery, metabolism, and efficacy. Furthermore, the data produced by animal models generally consist of measurements of fecal bacterial load, biopsies, and reports of animal morbidity and mortality, with little information gleaned about the bacterial reaction to the host environment at early time points after inoculation. As a result, the application of *in vitro* platforms has become more prevalent, with different systems used for studying microbe-molecule, microbe-microbe, microbe-microbiome, and microbe-host interactions.

## IN VITRO MICROBE-MICROBE, MICROBE-MICROBIOME, AND MICROBE-HOST PLATFORMS

Different *in vitro* platform designs stem from interest in probing distinct aspects of infection. Bacterial fermentation models are the most effective in ecological studies related to microbe-microbe or microbe-microbiome interactions. In *C. difficile* research, fermentation-based platforms are often used to model the effects of the gut microbiota on *C. difficile* metabolism and survival. Depending on the research question asked, batch fermentation or continuous single/multistage or microbe-host co-culture configurations can be used to reach an adequate balance between the necessary level of physiological relevance and accessibility of the platform (127, 128). For more detail on such *in vitro* modeling in *C. difficile* research, readers should consult an excellent recent review on the topic (128).

**Fig 2:**
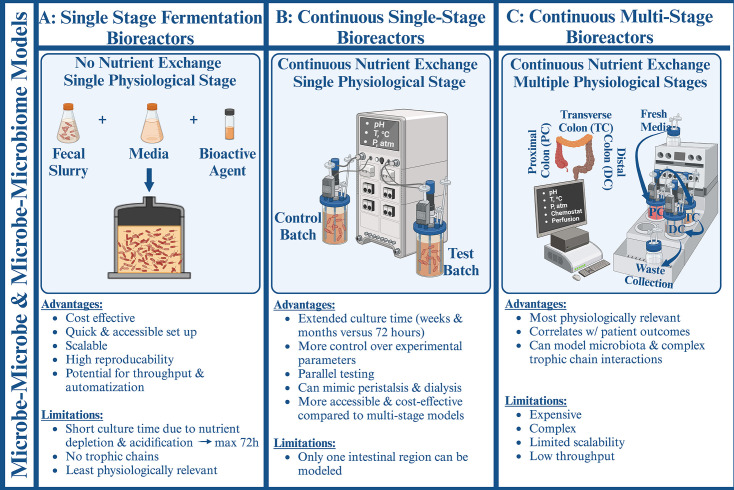
*In vitro* study models in *C. difficile* research probing microbe-microbe and microbe-microbiome interfaces. (**A**) Single-stage closed bioreactors offer accessible culturing of defined or complex microbial communities for up to 72 hours. (**B**) Continuous single-stage bioreactors allow nutrient input and waste product removal, necessary to establish steady-state bacterial communities. (**C**) Multichambered continuous-feed bioreactors model the physiologically distinct regions of the gastrointestinal tract, providing a platform for probing complex bacterial trophic chains. Created with BioRender.com

Single-stage fermentation bioreactors (SSFB), or simply batch fermentation bioreactors (Fig. 2A), utilize a single enclosed reaction vessel inoculated with a bacterial strain or a fecal sample. The resultant microbial culture continues to ferment in an anoxic environment at a controlled temperature for up to 72 hours, during which samples can be extracted for analysis (129). Batch fermentation is a simple, fast, relatively cheap, scalable, and reproducible method for probing transcriptional and/or metabolic activity as well as bacterial growth in response to bioagents and/or other bacterial species (128). As a result, batch fermentation represents an important preliminary step in testing novel bioactive agents and/or microbiome-based treatment strategies.

The first *C. difficile* fermentation model, developed by Borriello and Barclay in the mid-1980s, was essential for characterizing *C. difficile* growth kinetics and population dynamics mediated by the host gut microbiome (57, 130). Batch fermentation using fecal emulsion from animals with antibiotic-induced CDI was used to study the effects of selected antimicrobials on *C. difficile* growth, toxin production, and colonization resistance (57, 70, 131–134). This platform allowed a strain-dependent characterization of *C. difficile* sporulation and growth kinetics, important for epidemic strains (135). *In vitro* fermentation was utilized for probing *C. difficile* growth and sporulation rates in fecal emulsions collected from young children, contributing to our current understanding of the role asymptomatic children play in the transmission of this pathogen (131). This simple approach for probing *C. difficile* dynamics played a major role in important discoveries. For example, early batch fermentation studies uncovered the phenomenon of *C. difficile* colonization resistance mediated by the innate microbiome of the host. As a result, early fermentation experiments that facilitated the observation of this phenomenon laid the conceptual groundwork for developing modern microbiome-restorative CDI treatment strategies (136, 137). Recently, preliminary work with *Bifidobacterium* bacteriophage cocktail therapy utilized a batch fermentation approach to demonstrate the clearance of *C. difficile* in fermentation vessels containing patient-derived microbiome supplemented with *B. longum* and *B. breve* cultures (137). Similarly, it has been found that bacterial bile salt hydrolase activity, produced from microbial communities formed after administration of FMT, suppressed *C. difficile* germination in batch cultures (138).

Unfortunately, the extended culture times necessary for probing the long-term effects of restorative CDI treatments are incompatible with batch fermentation methodology. This is primarily due to rapid nutrient depletion and medium acidification, which limit the duration of a batch fermentation experiment to 72 hours (129). However, continuous-feed single-stage bioreactors (CSSB, Fig. 2B) can be used to expand the temporal experimental constraints associated with medium depletion. In these studies, fresh medium is continuously supplemented into the bioreactor chamber as waste is filtered out. Operational features, including the continuous nutrient-waste exchange, pH and temperature regulation, as well as atmospheric control, extend microbial culture time to weeks and even months (139). This allows experiments to continue for longer durations, enabling longitudinal modeling of how microbial communities adapt to perturbations such as the onset and end of antibiotic treatment. Continuous-stage bioreactors have been utilized to probe various aspects of *C. difficile* dynamics, including the effects of the environment, competition from innate microbiota, as well as the effects of bioactive agents (128). The main advantage of the CSSB platforms is their ability to establish complex steady-state bacterial communities while maintaining the accessibility of the experimental design (140). Depending on the goals of the study, CSSB design can be adapted to increase throughput or scalability. For example, multiple fecal microbial communities were grown in small volumes in minibioreactor arrays (MBRA) to investigate the competitive fitness of various CDI strains (141). A similar approach was used to demonstrate the inhibitory effect of polyphenols on *C. difficile* colonization resistance (142) and the synergistic behavior of *F. nucleatum* in forming a multi-species biofilm with *C. difficile* (143). However, in all configurations, continuous single-stage bacterial culturing models a single colonic region, which eliminates the potential for forming trophic chains characteristic of the entire colonic microbiome.

Microbial fermentation in proximal, transverse, and distal regions of the colon varies due to differences in pH and nutrient availability (144). Therefore, a continuous multi-stage (CMS) bioreactor system (Fig. 2C), reflective of the environment from all three colonic regions, represents the most physiologically relevant design among the *in vitro* microbe-microbiome platforms. The resultant trophic chains are not only well established but reflect the complexity of trophic chains formed from bacterial communities, characteristic of specific regions of the lower intestine. The multi-stage modification was originally introduced in the late 1980s and was later validated by chemical and bacteriological measurements against colonic luminal contents of human sudden death victims (145). This fermentation platform consists of three reaction vessels with distinct physiochemical parameters. Sterile, anaerobic media is supplied into the first reservoir mimicking the proximal colon (pH 5.5), which sequentially feeds the second (pH 6.2), and third (pH 6.8) reservoirs, modeling nutrient transit from the proximal through transverse to distal colonic regions. Further modifications can be made to recapitulate the environment of the entire GI tract with two more vessels, mimicking the stomach and the small intestine (144). Scientific knowledge related to region-specific nutrient absorption and bioavailability of bile salts, kinetics of gastric and pancreatic juices, as well as peristalsis, is used to enable tight control over the flow rate, temperature, pH, and retention in the system. Furthermore, continuous multi-stage platforms enable a steady-state maturation of a microbiome and formation of trophic chains, comparable with *in vivo* data in population diversity and metabolic profiles (145). As a result, this design represents the most physiologically relevant *in vitro* fermentation platform available today for modeling CDI.

In contrast to *in vivo* models, CMS platforms offer greater control over drug delivery and frequent sampling of microbial dynamics. Consistent with clinical and *in vivo* data, antibiotic exposure of human-derived microbiota in CMS bioreactor systems results in *C. difficile* spore germination and toxin production, making this design suitable for modeling disease and recurrence as well as probing treatment methods (146, 147). As a result, CMS fermentation is most commonly used in CDI research to characterize the efficacy of novel *C. difficile*-specific treatment methods and/or assess the potential of a given antibiotic to cause the disease (148). In addition to pre-clinical applications, CMS models are often used to probe the population dynamics associated with CDI. Modeling of multi-regional trophic chains allows probing various aspects of C. *difficile* colonization dynamics, resistance, virulence, and biofilm (27, 149). Although CMS models present the most relevant noninvasive alternative to *in vivo* research, this approach is complex and expensive, and requires extensive investment of resources, training, and scrupulous control by the operator, which makes scaling up difficult. Furthermore, CMS platforms focus on modeling the microbe-drug, microbe-microbe, or microbe-drug-microbiome dynamics of CDI, excluding host-mediated factors from the experimental design.

**Fig 3:**
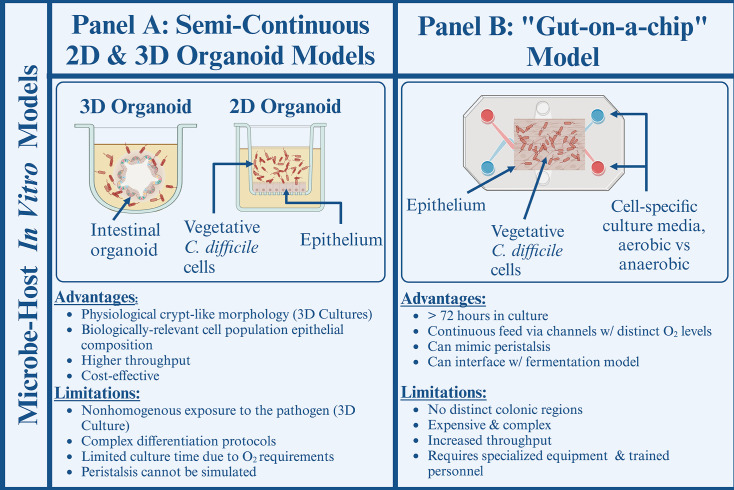
*In vitro C. difficile* study models probing the microbe-host interface. (**A**) Organoids derived from human stem cells can mimic biologically relevant tissue architecture (3D), exhibit adequate epithelial cell composition, and are relatively cost-effective. (**B**) Gut-on-a-chip platforms increase co-culture time with the anaerobic pathogen by providing a continuous, medium supply, segregating based on the cell-specific oxygen requirements. Created with Biorender.

The host-pathogen interface is difficult to add to existing *in vitro* platforms due to the practical challenges associated with co-culturing cells exhibiting drastically different physiological oxygen demands. Currently, the application of three-dimensional (3D) organoids or two-dimensional (2D) epithelial monolayers (Fig. 3A) is the option for probing interactions between mammalian epithelia and *C. difficile* cells *in vitro*.

Three-dimensional spheroids derived from induced pluripotent stem cells offer a high degree of physiological relevance, including a complex epithelium containing both epithelial and mesenchymal cells, as well as the presence of a compartmentalized anoxic environment comparable with the intestinal lumen. The outer surface of the spheroid is exposed to an oxygenated medium, satisfying the oxygen demands of the mammalian cells, while the interior is anaerobic enough to maintain the proliferation of *C. difficile* culture injected into the luminal space. The interiors of these enclosed spheroids contain well-defined crypts, morphologically resembling the tissue architecture of the intestinal epithelium*,* and can be used for studying host-pathogen dynamics and biofilm formation (143, 150, 151). Spheroids have been successfully used to model *C. difficile*-induced enteritis, where a combination of live microscopy and quantitative PCR techniques demonstrated variations in toxin-binding sensitivity in different cell lines (152). Similarly, organoids were used to evaluate the effects of *C. difficile* toxins A (TcdA) and B (TcdB) on the cytoskeletal integrity of the epithelium based on microRNA profiling (41). Despite reporting promising results, 3D culturing in general is not commonly used in the field due to practical limitations. These models are extremely difficult to scale up and have little potential for high-throughput work. In addition, complex 3D architecture also complicates live microscopy and necessitates the use of expensive, specialized equipment, inaccessible to many research teams. More importantly, the 3D morphology of the spheroids contributes to uneven exposure of mammalian cells to the given bacterial load. Cells located in the luminal section of the spheroid are often shielded from microbial exposure and require mechanical disruption to complete the inoculation. This translates into issues with data analysis and reproducibility for sequencing-based studies, as it is impossible to differentiate basolateral and luminal cells.

Organoid-based two-dimensional cultures are often used to address some of the accessibility and experimental design issues associated with both 3D cultures. This approach utilizes patient-derived cellular monolayers stabilized on a flat, coated surface, which ensures homogeneous exposure of the monolayer to treatment and can be compatible with microscopy. Transwell platforms are commonly used for host-microbe co-culturing, where the epithelial monolayer is submerged in a static medium with an established oxygen gradient. As with sealed microbial bioreactors, there is a finite co-culturing time possible before cytotoxicity takes place. Promisingly, recent modifications of the transwell-based approach have demonstrated a successful 48 hours of co-culturing of a patient-derived epithelial monolayer with oxygen-sensitive *Faecalibacterium prausnitzii* (151). This monolayer developed a crypt-like architecture and maintained a mucosal barrier, which is lacking in 3D organoids. Although relatively accessible, this study model relies on the separation of the aerobic and anaerobic environments, limiting physical contact between the host and the microbe. Furthermore, real-time probing is challenging and can be achieved primarily via the sampling of co-culture medium for bacterial toxins or mammalian metabolites. More complex and resource-intensive configurations are needed to address limitations related to a lack of physical interactions between the host and the microbe, as well as real-time probing.

Some of these limitations are addressed by the use of an epithelial vertical diffusion chamber (EVDC) to continuously replenish an oxygen gradient on either side of the porous polyester scaffold of a transwell culture insert. The Unnikrishan team has recently validated this approach for 2D and 3D co-culture of *C. difficile* with mammalian cells (153). Mixed monolayers of Caco-2 and HT29-MTX goblet cells cultured within the insert are positioned between two compartments inside the EVDC and sealed with epithelial voltage clamps, which monitor the monolayer integrity based on the measurement of transepithelial resistance (TEER). Dual chamber design allows the epithelial monolayer to be simultaneously exposed to aerobic and anaerobic environments, which are maintained by delivering oxygen-rich and oxygen-depleted gas mixes through two independent inlets. Oxygen diffusion occurs through the porous scaffold to the basolateral side of the monolayer, while the apical side is inoculated with live *C. difficile* under anaerobic conditions. The platform can be modified to support multi-layer and 3D co-cultures, where Caco-2/HT29-MTX monolayers are grown atop human fibroblasts, effectively mimicking the structural organization of the colonic tissue. This approach improves the limitations associated with conventional Transwell co-culture by enabling the physical interactions between the bacterial and the host cells, as well as monitoring the integrity of the host monolayer in real time. EVDC has been used to characterize the adherence of *C. difficile* to mammalian cells during early infection, as well as *C. difficile* population growth in the presence of commensal *Bacteroides dorei*. More recently, the E-VDC platform was utilized for analyzing the bacterial and host transcriptomic profiles using RNA-sequencing (154). While the EVDC is an incredibly sophisticated model of the host-pathogen interface, it requires complex and costly materials inaccessible to many research groups. In addition, the real-time TEER measurement reports strictly on the integrity of the host monolayer, which can be affected by various factors, including bacterial infection. Therefore, a lack of direct representation of the host-bacterium interface during the co-culture poses the main limitation of this approach.

Microfluidic intestine-on-chip (MIC) platforms are another example of *C. difficile* host-pathogen co-culture platforms compatible with real-time analysis. This approach resolves the oxygen demand incompatibility by culturing intestinal epithelial cells on a porous scaffold sandwiched between two channels characterized by distinct oxygenation levels (155). As with the EVDC, perfusion of O_2_-depleted medium above the epithelium supports microbial growth, including that of obligate anaerobes, while the O_2_-rich media flowing below the cellular scaffold delivers oxygen and nutrients to the mammalian cells and removes waste. As a result, an epithelial monolayer can continue to grow for days to weeks, facilitating the formation of steady-state microbial communities, necessary for probing various aspects of the microbiome-pathogen-host interface (155, 156). Further modifications can be made to allow for real-time measurement of the pH and oxygen levels throughout the experiment or mimic the *in vivo* peristalsis (157). Recently, a hypoxic MIC platform was used to demonstrate the novel role of *C. difficile* binary toxin (CDT) in enhancing colonization and persistence of the infection by forming biofilm-like microcolonies resistant to vancomycin (158). This study validated the use of MIC platforms as a CDI model and demonstrated the formation of microcolonies consistent with *in vivo* rodent models utilized in the same study. Application of both 2D and 3D mammalian culture in CDI research offers a significant degree of physiological relevance, especially when conducted using the MIC platform. Unfortunately, widespread utilization of these methods is difficult to achieve due to the high cost, requirement for highly trained personnel, sophisticated equipment, and extensive protocols. Furthermore, neither transwell plate nor gut-on-a-chip models are compatible with live microscopy, eliminating the potential for collecting and analyzing data with single-cell resolution. Consequently, *in vitro* research modeling the *C. difficile* host-pathogen interface at the cellular level is limited.

## SIMPLIFYING THE MICROBE-HOST INTERFACE

None of the co-culture platforms described thus far are readily available to groups lacking access to highly specialized equipment and many depend on access to primary cells obtained from patient samples. Naturally, researchers gravitate toward straightforward and cost-effective experimental design. This, at least in part, explains the prevalence of bulk screening and population dynamics research compared to work targeting the host-pathogen interface.

**Fig 4:**
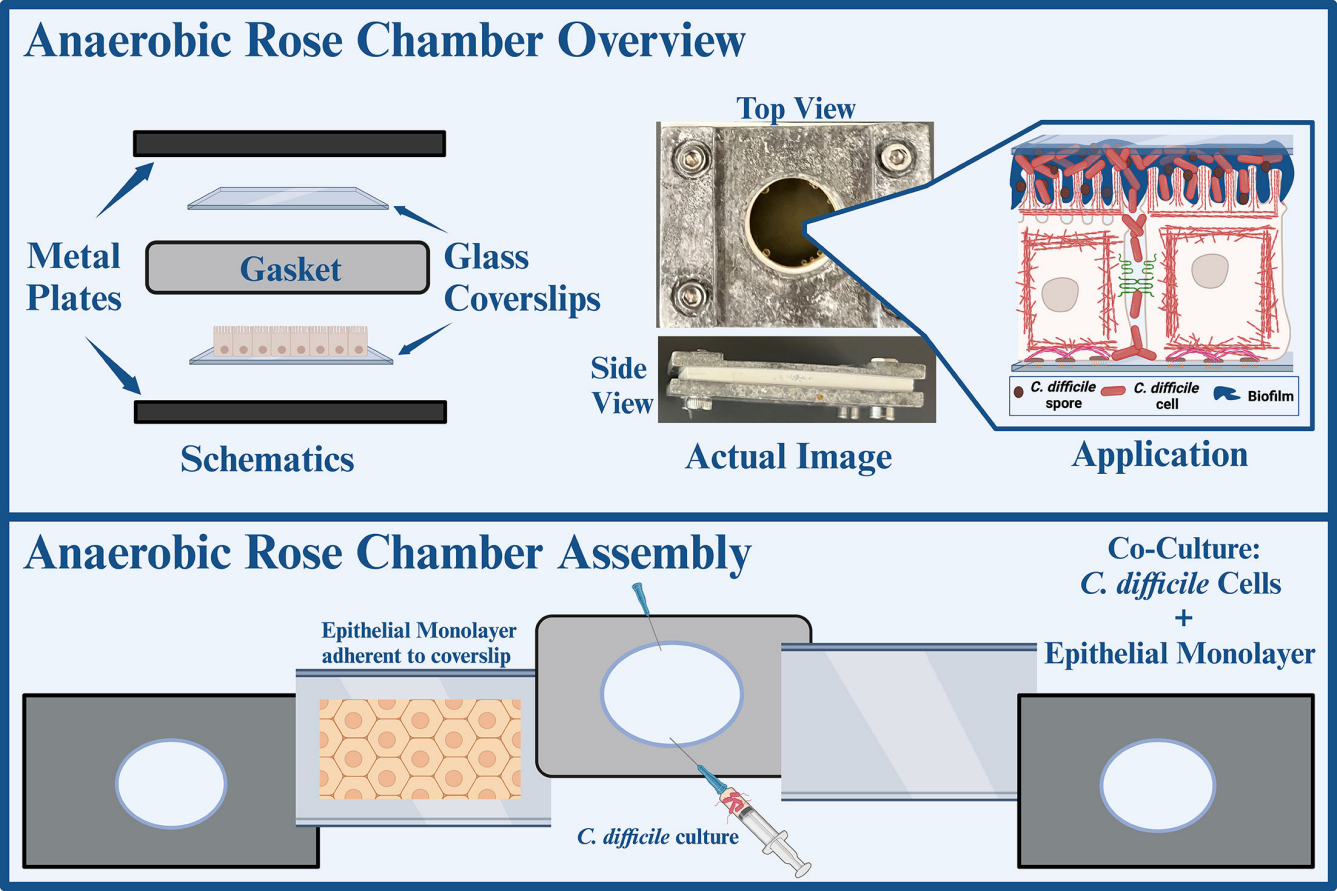
Anaerobic rose chambers offer an opportunity for co-culturing obligate anaerobes with mammalian cells. Shown in the top panel is the overview of the platform, including the chamber parts and an actual image of the assembled chamber containing a 48 hour old *C. difficile* culture. The bottom panel visualizes the assembly.

Relatively fast and easy batch fermentation models contributed to many important discoveries and the development of treatment strategies. More recent modifications to address limited experimental time and over-simplification of the bacterial habitat led to the development of the CSSB and CMS platforms, which allowed additional findings to complement those obtained with more accessible methods. The findings from simpler models have been very important in shaping the design of more complex experiments, just as *in vitro* results are crucial preliminary data for the design of *in vivo* experiments. The currently available host-microbe co-culture models rely on complex methodology and specialized equipment, and there are few accessible techniques to provide the real-time single-cell analysis that would be important preliminary data for such studies. Anaerobic rose chamber imaging has the potential to fill that niche, serving as an equivalent of batch fermentation in single-cell *C. difficile* research (Fig. 4).

**Fig 5:**
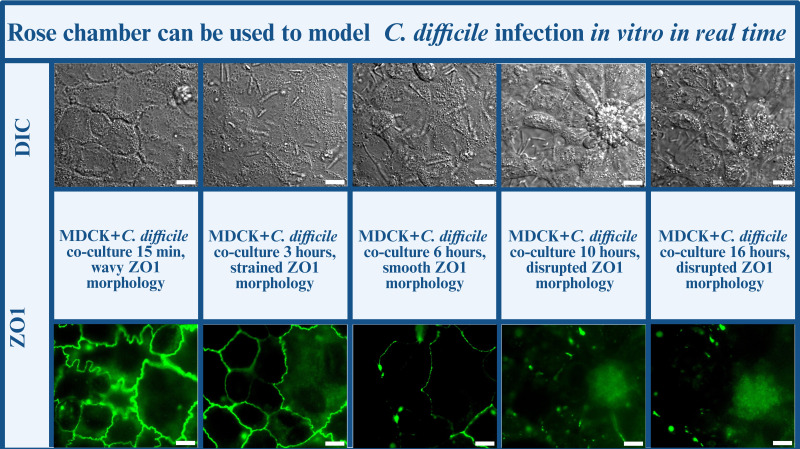
Validation of the MDCK cell line as a suitable *in vitro* host model for probing host-pathogen interactions with *C. difficile in vitro*. The panel demonstrated the progression of the *C. difficile* infection *in vitro* with mammalian MDCK cell monolayer and its effect on the integrity of cell-to-cell junctions. MDCK junctions were visualized based on the ZO-1 GFP fluorescent tag, illustrating the gradual disruption of cellular architecture. Images provided by Dr. David Courson. Figure created with Biorender.

A rose chamber is a multi-use device, consisting of two metal plates, a rubber gasket and coverslips that can be assembled into a chamber as shown in Fig. 4. A hole cut in the middle of the rubber gasket creates a space for a small volume of culture, which can contain aerobic medium or be equilibrated to an anaerobic environment and filled with anoxic medium. Glass coverslips sandwich the hole in the middle of the gasket to seal the space for the bacterial culture while keeping it optically compatible with live bright field, phase contrast, and fluorescent microscopy. Metal plates pulled to each other by screws press the coverslips to the gasket and create a liquid-proof seal with minimal gas exchange with the environment. The assembled chamber is filled with bacterial culture through a syringe inserted into the rubber gasket, with a second needle allowing the ejection of gas as the central cavity is filled. After the chamber is filled, the needles are removed and the rubber gasket seals the residual holes. Anaerobic rose chambers have previously been utilized to characterize nutrient-mediated motility of individual live *C. difficile* cells using a standard inverted microscope (159). Furthermore, rose chambers can be adapted to study *C. difficile* interactions with mammalian epithelia by culturing a polarized monolayer of hypoxia-tolerant epithelial cells, such as Madine-Darbey canine kidney cells (MDCK) (160, 161) on one of the coverslips used to assemble the rose chamber (Fig. 5). Here, live fluorescent microscopy images demonstrate ZO-1 tight junction morphology fluorescently labeled in MDCK cells comprising a monolayer inside the rose chamber in various environmental conditions. Of note, normal ZO-1 morphology is visualized in aerobic conditions, anaerobic conditions, and in culture with live *C. difficile*. Figure. 5 contains a selection of representative images demonstrating the effect of *C. difficile* co-culture on an MDCK monolayer by tracking the integrity of ZO1 cell-to-cell junctions for 16 hours. Live microscopy allows for real-time, single-cell analysis of ZO1 integrity, and captures unique events in the host-pathogen interface. Specifically, disruption of the wave-like morphology associated with the arrangement of ZO1 in the cell wall followed the change in fluorescence pattern. These images demonstrate the potential for quantitative analysis of changes in fluorescence profiles of the tagged cellular targets (MDCK ZO1 junction protein in this case) over time. This approach captures unique cellular events that would have remained unnoticed otherwise. For example, individual bacterial cells tend to attach to cell-cell surface of the host monolayer rather than the middle of cell bodies (Fig. 5). As adherent bacterial biofilms on mammalian tissue are typically observed after formation rather than during, this finding could potentially inform more detailed future studies on the regulation of bacterial adhesion to and penetrance into epithelial monolayers. Furthermore, the real-time analysis provides a temporal characterization of the cell death events associated with bacterial infection, not evident from end-point analysis of ZO1 morphology. Future work could involve multipexing with multi-reporter systems, where fluorescent tags with distinct fluorescent profiles can be utilized to probe multiple cellular targets within the host or in the host and the pathogen. Therefore, anaerobic rose chamber imaging can fill the gap in the field and be widely used as a simple, accessible, and user-friendly tool for real-time probing of the host-pathogen interface to characterize various stages of *C. difficile* pathogenesis.
